# Maternal Administration of Aripiprazole Impedes the Appendicular Skeletal Growth of Rat Fetuses: A Teratological and Histomorphometrical Assessment

**DOI:** 10.3390/biomedicines14061294

**Published:** 2026-06-06

**Authors:** Bhagath Kumar Potu, Mariwan Husni, Wael Amin Nasr El-Din, Abdel Halim Salem, Aisha Rashid, Amer Almarabheh, Raouf Abdelrahman Fadel

**Affiliations:** 1 Department of Anatomy, College of Medicine and Health Sciences, Arabian Gulf University, Manama 329, Bahrain; waela@agu.edu.bh (W.A.N.E.-D.); ahaleemfd@agu.edu.bh (A.H.S.); aishar@agu.edu.bh (A.R.); raoufre@agu.edu.bh (R.A.F.); 2 Department of Psychiatry, Northern Ontario School of Medicine (NOSM) University, Thunder Bay, ON P7B 5E1, Canada; mariwan.husni@nosm.ca; 3 Department of Human Anatomy and Embryology, Faculty of Medicine, Suez Canal University, Ismailia 41522, Egypt; 4 Department of Family and Community Medicine, College of Medicine and Health Sciences, Arabian Gulf University, Manama 329, Bahrain; amerjka@agu.edu.bh

**Keywords:** aripiprazole, prenatal, appendicular skeleton, ossification, rat fetuses

## Abstract

**Background/Objectives**: A third-generation atypical antipsychotic drug, aripiprazole, is known to cross the placental barrier and pose negative consequences on placental growth and the normal development of the fetus. Although a few studies demonstrated these debilitating effects of aripiprazole, its skeletal effects remain unexplored. Therefore, this study was undertaken to evaluate the impact of prenatal aripiprazole exposure, administered at three different dose levels, on the ossification of the appendicular skeleton in 20-day-old rat fetuses. **Methods**: Forty pregnant Sprague–Dawley rats (*n* = 40) were assigned to four groups: control and three aripiprazole-treated groups receiving 3 mg/kg (LDA), 6 mg/kg (HDA), and 12 mg/kg (DHDA) daily from gestational days 6–19. Fetuses were delivered on gestation day 20, weighed, and processed for skeletal evaluation using Alizarin Red S staining. Ossification patterns of metacarpals, metatarsals, hip bones, long bones of the forelimb and hindlimbs from a total of 151 fetuses were analyzed and categorized as complete, delayed, or absent. **Results**: Aripiprazole exposure induced a dose-dependent reduction in the number of completely ossified skeletal bony centers (*p* < 0.01) with a highly significant reduction in the length of ossified portions of the long bones (*p* < 0.001). Histomorphometric analysis of Von Kossa-stained fetal femur sections revealed a significant decrease in the thickness of ossified cortical and trabecular bone with a statistically significant reduction in the length of hypertrophied chondrocytes of the growth plate cartilage in the aripiprazole-treated groups (*p* < 0.05). **Conclusions**: Prenatal exposure to aripiprazole leads to dose-dependent skeletal growth restriction and delayed ossification of the appendicular skeleton in rat fetuses. Future investigations should focus on the molecular mechanisms and consequences related to the prenatal impact of aripiprazole.

## 1. Introduction

Long-term usage of antipsychotic drugs (APDs) is associated with numerous side effects, such as akathisia, dystonia, tardive dyskinesia, weight gain, adverse cardiovascular events, type 2 diabetes [1], decreased bone mineral density (BMD), and increased fracture risk possibly due to lifestyle factors of psychiatric disorders such as a reduced physical activity, smoking, poor dietary habits, and alcohol consumption in addition to genetic and biological factors [2,3,4]. A large-scale study from the Manitoba Bone Density Program database with 68,730 participants, 90.6% women, using APDs revealed a 1.4-fold increased risk of osteoporotic fractures and a 2.1-fold increased risk of hip fracture compared with the non-users [5].

A third-generation atypical antipsychotic drug (AAPD), aripiprazole, has been introduced quite recently and proven to be successful in treating positive, negative, and cognitive symptoms [6]. APD usage during pregnancy has doubled during the last decade. However, their safety for the developing fetus and the potential risks of congenital malformations are not known on a larger scale [7]. A large-scale study conducted by Huybrechts and colleagues, including 1.34 million pregnancies, revealed a higher risk for congenital malformations among women treated with typical antipsychotics than with atypical antipsychotic drugs (AAPDs) [7]. The recently published literature provided reassuring data about the use of aripiprazole during pregnancy, peripartum, and even lactation [8]. However, understanding the “double-edged sword” effect of any medication on the mother and fetus administered during pregnancy, clinicians must meticulously weigh the risks and benefits of prenatal drug use during intrauterine development, as intrauterine organ development is vulnerable to adverse environments, and many different kinds of drugs have been shown to damage the development of the organs [9].

The majority of the APDs act on dopamine, serotonin, and adrenergic receptors, which are mostly confined to the brain, but recent research findings have revealed that they are even present in osteoblasts and osteoblasts [10]. The osteoblasts are known to facilitate bone formation, while the osteoclasts are responsible for resorbing the bone [11]. Any imbalance in the expression of dopamine, serotonin, and adrenergic receptors on bone cells may ultimately lead to bone loss [12]. In light of this emerging experimental evidence, we assume that the AAPDs, such as aripiprazole, have more teratogenic effects on the skeletal ossification of developing fetuses when mothers are treated with this drug during pregnancy. A few studies from our lab suggested that aripiprazole could impair the normal placental developmental growth [13] and could even cross the placental barrier and enter the fetal circulation due to its smaller molecular weight and hamper the normal development of offspring with a significant increase in embryo-lethality [14].

Although a study describing the negative impact of aripiprazole on bone health in adolescents and young adults by inhibiting the osteoblast cell viability [15], studies on the effects of aripiprazole in fetuses, particularly on the number, pattern of the ossification centers (complete, delayed, or absent), and length of the ossified bone in the appendicular skeleton are not available in the literature. Therefore, we have undertaken this study to explore the effects of aripiprazole on the ossification of bony centers, length, and microarchitecture of the fetal appendicular skeleton.

## 2. Materials and Methods

### 2.1. Animals

Female Sprague–Dawley rats, 2 months old (Charles River Laboratories, Margate, Kent, UK), weighing 150–200 g (*n* = 40) were used in this study. This study was conducted upon obtaining ethical approval from the Research and Ethics Committee (REC) of the College of Medicine and Health Sciences (CMHS), Arabian Gulf University (AGU) (Approval no: E019-PI-11/20). Rats were housed at the central animal research facility of AGU in sanitized stainless-steel cages (Techniplast TM, Buguggiate, Italy) under standard conditions of temperature (23 °C ± 2 °C) and lighting (12 light/dark cycles). All the rats were allowed free access to food and drinking water in accordance with the regulations of the REC of CMHS, AGU.

### 2.2. Experimental Design

Following 10 days of acclimatization, the female rats were mated with a healthy, fertile male rat as per standard protocol to achieve pregnancy. Pregnancy was confirmed by evaluation of the vaginal smear the next morning with the presence of spermatozoa, and that day is considered the first day of gestation. After this, a total of 40 (*n* = 40) healthy pregnant rats were randomly divided into four groups of 10 rats each (*n* = 10). Group I, the control group, received distilled water, group II, the low-dose aripiprazole (LDA) group, group III, the high-dose aripiprazole (HDA) group, and group IV, the double high-dose aripiprazole (DHDA) group received 3, 6, and 12 mg/kg body weight doses of aripiprazole; respectively from the 6th to the 19th day of pregnancy, after being dissolved in distilled water and the route of administration was by gavage. The doses were calculated according to the weight of the dams and adjusted to the human doses as described in our previous study [14] using a standard pharmacological “human to animal dose” conversion formula [16].

The calculation of the precise dose administered to the rats adhered to the following equation:

Rat dose (mg/kg) = Human Equivalent Dose (mg/kg) × Km ratio of rat

Km ratio conversion factor = 6.2 [16].

In the current study, we converted the maximum recommended human therapeutic dose (MRTD): 30 mg/day to a rat dose and utilized it as the low dose of aripiprazole (LDA). To investigate the upper limits of risk, we included two higher-dose groups that exceeded the MRTD after conversion to a rat dose. The high-dose of aripiprazole (HDA) was twice the LDA, and the double high-dose of aripiprazole (DHDA) was quadruple the LDA. Based on an assumed average woman’s weight of 65 kg for calculating the human equivalent dose, the three experimental aripiprazole doses utilized in the rat model were: 3 mg/kg/body weight (LDA), 6 mg/kg/body weight (HDA), and 12 mg/kg/body weight (DHDA).

At the end of treatment, i.e., on day 20, pregnant rats were sacrificed under CO_2_ inhalation. A cesarean section was performed to collect the fetuses. The membranes of the fetuses were stripped and detached from their placentas, and then the fetal weight (FW) was recorded.

#### 2.2.1. Alizarin Red S Staining of the Fetuses

As previously outlined in our lab protocols [17], half the number of fetuses from each mother underwent skeletal staining with Alizarin Red S. The remaining half were fixed in Bouin’s solution for studying histological parameters. For Alizarin Red S staining, fetuses from the control, LDA, HDA, and DHDA were eviscerated and skinned. Skeletal staining was performed on the eviscerated and skinned fetuses using a standard protocol described by Dawson [18]. In brief, the process began with the fetuses being immersed in absolute ethanol for 5 days, followed by a transfer into a 1% potassium hydroxide solution for 1–2 days to clear the bones. Following this step, fetuses were subsequently transferred to a fresh solution of 0.5% potassium hydroxide, to which a few drops of Alizarin Red S staining solution were added and left for 1–2 days. Then, the fetuses were moved to concentrations of 30%, 50%, and 70% glycerin, respectively, at 2–3 day intervals and finally stored in 100% pure glycerin containing a few pieces of thymol to prevent fungal growth.

#### 2.2.2. Scoring of the Ossification Centers

Following a previously published protocol by Nash and Persaud, a skeletal scoring chart was designed to show the ossification centers observed on gestational day 20 in Sprague–Dawley rat fetuses [19]. A Zeiss Stemi 2000-C dissecting microscope (Zeiss, Oberkochen, Germany) was used to analyze the appendicular skeletal ossification centers in each fetus. Ossification patterns were classified as either complete, delayed, or absent. Completely ossified centers were indicated by strong staining, while delayed ossification showed weak staining, small, or dumbbell-shaped ossified centers.

#### 2.2.3. Measurement of Ossified Bones

Alizarin Red S-stained limbs were photographed to measure the ossified length of the forelimb and hindlimb bones. Lengths of the ossified humerus, radius, ulna, femur, tibia, and fibula were measured using a Zeiss Stemi 2000-C dissecting microscope (Zeiss, Oberkochen, Germany). The mean total lengths of the ossified bones stained with Alizarin Red S in the control and treated groups were calculated.

#### 2.2.4. Histomorphometric Analysis

The whole femurs were isolated from the pups, dissected free of soft tissue, and fixed with 10% formalin, pH 7.4, and stored at room temperature for 24 h. The whole femurs of fetuses from different groups were dehydrated in a graded series of alcohols, embedded in paraffin wax, and sectioned (4 μm thickness) longitudinally on a rotary microtome (Leica, Wetzlar, Germany) and processed for Von Kossa staining to analyze the thickness of cortical, trabecular bone, and hypertrophied chondrocytes as per standard protocols [20,21]. During the embedding process, care was taken to ensure that all femurs were oriented in the same direction to minimize differences in the angles at which the bones were sectioned.

#### 2.2.5. Von Kossa Staining

**Figure 1:**
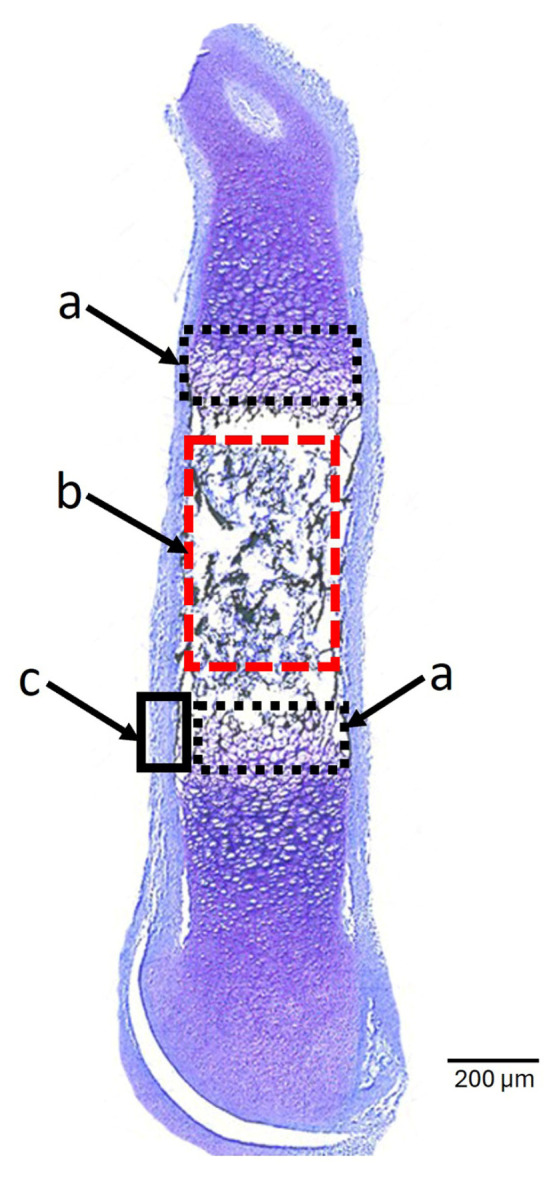
Von Kossa-stained femur sections showing the quantified areas from different groups. Dotted black square area (a) showing the hypertrophied zone of growth plate cartilage measured; dotted red square area (b) showing the thickness of trabecular bone measured in the diaphysis, and black square area (c) showing the cortical bone thickness measured.

Formalin-fixed bone sections (4 μm thickness) were processed with a 5% silver nitrate solution in bright sunlight (or in front of a 60-watt lamp) for 3 h or until the bone sections turned black. Thereafter, sections were processed through a 5% sodium thiosulfate solution for 5 min after rinsing the sections thoroughly in distilled water. Thereafter, slides were counterstained with a 0.5% Toluidine Blue solution for 2 min [21]. All the sections were processed uniformly to maintain consistency in the histomorphometrical analysis (Figure 1). The thickness of cortical bone and the optical density of the trabecular bone were measured using ImageJ software (version 1.53; Wayne Rasband NIH, Bethesda, MD, USA). In addition, the length of hypertrophic chondrocytes in the growth plate cartilage was also measured using ImageJ software in the upper and lower ends of the femur to understand the impact of aripiprazole on endochondral ossification and the extent of mineralization among the groups. The length of hypertrophic chondrocytes was determined by measuring their length in at least three areas of interest per slide.

To minimize experimental bias, the groups were blinded. From these blinded groups, scoring of the ossification centers, measurement of the ossified portion of long bones, and histomorphometrical analysis were independently evaluated by three investigators (RAF, BKP, and WANED). Randomization, allocation concealment, and blinding of the experimental groups were performed following a standard research protocol [22].

#### 2.2.6. Statistical Analysis

Quantitative variables were presented as means and standard deviations, whereas categorical variables were descriptively summarized using frequencies and corresponding percentages. All statistical analyses considered the dam as the experimental unit; therefore, the mean value of fetal parameters within each litter was used for group comparisons. The two-proportion Z-test was used to determine the difference between two proportions. The Pearson correlation coefficient was used to measure the relationship between two quantitative variables. The one-way analysis of variance (ANOVA) was used to compare the mean length of the ossified parts of the long bones, hypertrophied chondrocytes, and optical density of trabeculae with cortical bone thickness in different groups. The post-hoc tests using Dunnett’s, Bonferroni, and Tukey’s methods were used to determine the significance of pairwise mean differences in the above-mentioned parameters. Data were analyzed using the Statistical Package for the Social Sciences (SPSS), version 30 (Chicago, IL, USA), and Minitab Statistical Software, version 17 (Minitab, Inc., State College, PA, USA). A *p*-value less than 0.05 was considered statistically significant.

## 3. Results

### 3.1. Examination of the Ossified Centers of the Appendicular Bones

Ossification of the appendicular bones in Alizarin Red S-stained 20-day rat fetuses of the control and aripiprazole-treated groups is summarized in Table 1.

### 3.2. Effect of Aripiprazole on the Extent of Ossification in Metacarpals and Metatarsals

#### 3.2.1. Metacarpals

**Figure 2:**
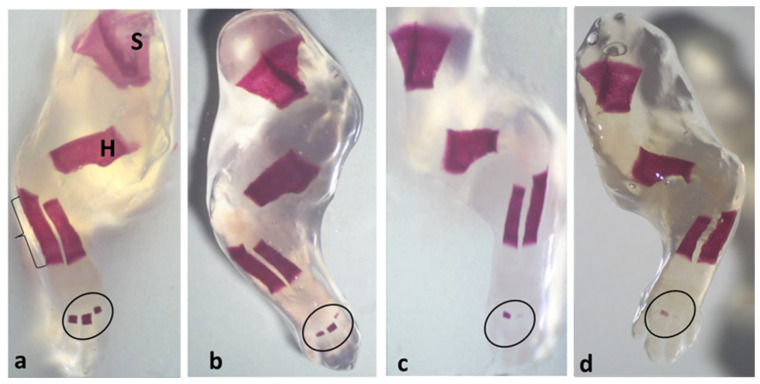
Circled areas showing the ossification patterns of the metacarpals of Alizarin Red S-stained 20-day rat fetuses in the (**a**) control, (**b**) LDA: low-dose aripiprazole, (**c**) HDA: high-dose aripiprazole, and (**d**) DHDA: double high-dose aripiprazole-treated groups. Of the 5 metacarpals, only 3 metacarpals (2nd, 3rd, and 4th of the circled areas) showed complete ossification in the control group (**a**). Such normal metacarpal ossification was seen delayed/absent (circled areas) in aripiprazole-treated groups in a dose-dependent manner (**b**–**d**). S: Scapula; H: Humerus; bracketed area: Ulna and Radius.

A total of 1510 metacarpals from 151 rat fetuses were examined from all the groups. It was noted that the complete ossification centers in the examined metacarpals were significantly reduced in the LDA, HDA, and DHDA-treated groups when compared to the control group (*p* < 0.01) and in DHDA compared to the HDA-treated group (*p* < 0.01). These similar patterns were even found among the absent and delayed ossification centers of the metacarpals in the LDA, HDA, and DHDA-treated groups when compared to the control group (*p* < 0.01) and between the HDA and DHDA-treated groups (*p* < 0.01). However, these patterns were not statistically significant between the LDA–HDA and LDA–DHDA groups (*p* > 0.05) (Figure 2 and Table 2).

#### 3.2.2. Metatarsals

**Figure 3:**
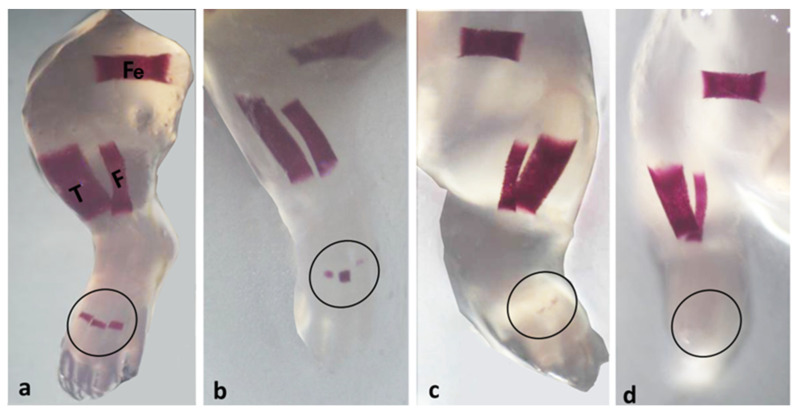
Circled areas showing the ossification patterns of the metatarsals of Alizarin Red S-stained 20-day rat fetuses in the (**a**) control, (**b**) LDA: low-dose aripiprazole, (**c**) HDA: high-dose aripiprazole, and (**d**) DHDA: double high-dose aripiprazole-treated groups. Of the 5 metatarsals, only 3 metatarsals (2nd, 3rd, and 4th of the circled areas) showed complete ossification in the control group (**a**). Such normal metatarsal ossification was seen as delayed/absent (circled areas) in the aripiprazole-treated groups in a dose-dependent manner (**b**–**d**). Fe: Femur; T: Tibia; F: Fibula.

A total of 1510 metatarsals from 151 rat fetuses were examined from all the groups. It was noted that the complete ossification centers in the examined metatarsals were significantly reduced in the LDA, HDA, and DHDA-treated groups when compared to the control group (*p* < 0.01). These similar patterns were even found among the absent and delayed ossification centers of the metatarsals in the LDA, HDA, and DHDA-treated groups when compared to the control group (*p* < 0.01). However, these patterns were not statistically significant between the LDA–HDA, LDA–DHDA, and HDA–DHDA groups (*p* > 0.05) (Table 2 and Figure 3).

### 3.3. Effect of Aripiprazole on the Length of Ossified Portions of the Long Bones of Forelimb and Hindlimb and Fetal Weight (FW)

#### 3.3.1. Humerus

A total of 302 humeri (78 Control, 76 LDA, 90 HDA, and 58 DHDA) from 151 rat fetuses were examined. It was noted that there was a significant reduction in the length of the ossified portion of the humerus in all treated groups compared to the control, with the greatest reduction observed in the DHDA-treated group. Furthermore, the DHDA-treated group showed a highly significant reduction when compared to the LDA and HDA-treated groups (*p* < 0.001) (Table 3).

#### 3.3.2. Radius

A total of 302 radii (78 Control, 76 LDA, 90 HDA, and 58 DHDA) from 151 rat fetuses were examined. It was noted that there was a significant reduction in the length of the ossified portion of the radius in all treated groups compared to the control, with the greatest reduction observed in the DHDA-treated group. Furthermore, the DHDA-treated group showed a highly significant reduction when compared to the LDA and HDA-treated groups (*p* < 0.001) (Table 3).

#### 3.3.3. Ulna

A total of 302 ulnae (78 Control, 76 LDA, 90 HDA, and 58 DHDA) from 151 rat fetuses were examined. It was noted that there was a significant reduction in the length of the ossified portion of the ulna in the HDA and DHDA-treated groups compared to the control group. However, no significant reduction was noted in the LDA-treated group when compared to the control. Moreover, the DHDA-treated group showed a highly significant reduction when compared to the LDA-treated group (*p* < 0.001) (Table 3).

#### 3.3.4. Femur

A total of 302 femurs (78 Control, 76 LDA, 90 HDA, and 58 DHDA) from 151 rat fetuses were examined. It was noted that there was a significant reduction in the length of the ossified portion of the femur in all treated groups compared to the control. Moreover, no significant difference was noted between the LDA and HDA-treated groups. On the other hand, the DHDA-treated group exhibited a highly significant reduction compared to the control, LDA, and HDA-treated groups (*p* < 0.001) (Table 3).

#### 3.3.5. Tibia

A total of 302 tibiae (78 Control, 76 LDA, 90 HDA, and 58 DHDA) from 151 rat fetuses were examined. It was observed that there was a significant reduction in the length of the ossified portion of the tibia in all treated groups compared to the control. Furthermore, no significant difference was noted between the LDA and HDA-treated groups. On the other hand, the DHDA-treated group exhibited a highly significant reduction compared to the control, LDA, and HDA-treated groups (*p* < 0.001) (Table 3).

#### 3.3.6. Fibula

A total of 302 fibulae (78 Control, 76 LDA, 90 HDA, and 58 DHDA) from 151 rat fetuses were examined. It was observed that there was a significant reduction in the length of the ossified portion of the fibula in all treated groups compared to the control. Furthermore, no significant difference was noted between the LDA and HDA-treated groups. On the other hand, the DHDA-treated group exhibited a highly significant reduction compared to the control, LDA, and HDA-treated groups (*p* < 0.001) (Table 3).

Regarding the FW, there was a significant reduction in the HDA and DHDA-treated groups compared to the control (*p* < 0.05).

### 3.4. Effect of Aripiprazole on the Extent of Ossification in Hip Bones

**Figure 4:**
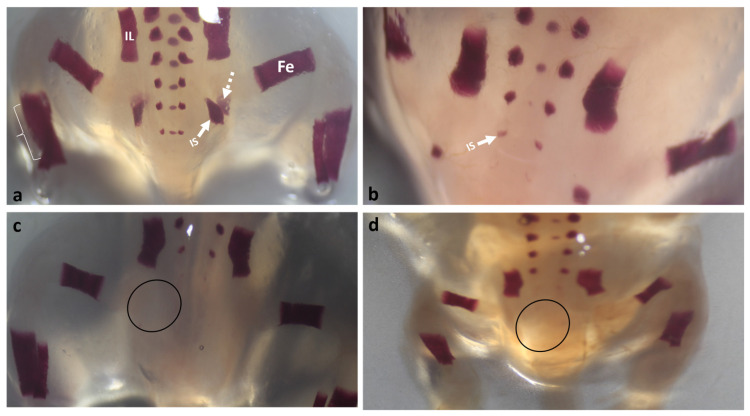
Ossification patterns of the hip bones of Alizarin Red S-stained 20-day rat fetuses in the (**a**) control, (**b**) LDA: low-dose aripiprazole, (**c**) HDA: high-dose aripiprazole, and (**d**) DHDA: double high-dose aripiprazole-treated groups. Control group (**a**) shows complete ossification of the ilium (IL) and ischium (IS) with incomplete ossification of the pubis (dotted white arrow). Such normal ossification of the ischium (IS) was delayed with absent pubis in the LDA group (**b**), and the ischium (IS)-pubis was seen absent (circled area) from the HDA and DHDA aripiprazole-treated groups in a dose-dependent manner (**c**,**d**). Fe: Femur; bracketed area: Tibia and Fibula.

Table 4 shows a significant decrease in the total number of completely ossified bony centers of the hip bones (6 centers/fetus) that was noted in the aripiprazole-treated groups when compared to the control group. A significant reduction in the complete ossification centers with a significant increase in the number of delayed/absent ossified bony centers of the ilium and ischium were noted in the LDA, HDA, and DHDA-treated groups when compared to the control group (*p* < 0.01) (Table 4 and Figure 4). However, such patterns were not seen in the ossification of the pubis.

### 3.5. Correlations Between FW and the Lengths of the Ossified Portions of the Long Bones of the Forelimb and Hindlimbs

Correlation between FW and the lengths of the ossified portions of the long bones of the forelimb and hindlimbs in all groups is presented in Table 5. In the control group, the Pearson correlation coefficient revealed statistically significant positive correlations between FW and humerus (r = 0.629, *p* < 0.01), radius (r = 0.537, *p* < 0.01), and ulna (r = 0.416, *p* < 0.05) when compared to all the other long bones of the LDA, HDA, and DHDA groups.

### 3.6. Effect of Aripiprazole on the Length of Hypertrophied Chondrocytes in the Growth Plate Cartilage of Fetal Femur

**Figure 5:**
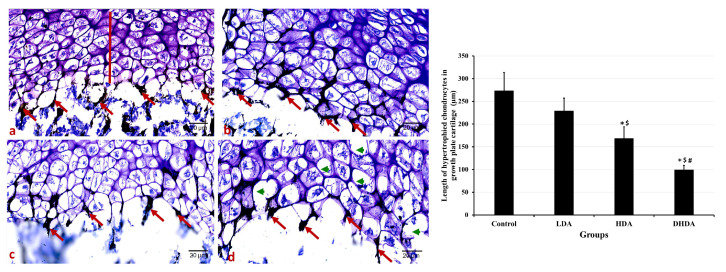
Von Kossa-stained longitudinal sections of fetal femur (**a**–**d**) showing the length of hypertrophied chondrocytes (µm) measured in the growth plate cartilage of the (**a**) control, (**b**) LDA, (**c**) HDA, and (**d**) DHDA-treated groups. Red vertical line indicating the length of hypertrophied chondrocytes measured (**a**). Red arrows show the mineralized bone in the primary spongiosa of all groups. Green arrowheads showing the inactive chondrocytes devoid of nuclei in the DHDA-treated group (**d**). Scale bar: 20 µm. Histogram values are expressed as Mean ± SD. * *p* < 0.05 vs. control group, $ *p* < 0.05 vs. LDA group, and # *p* < 0.05 vs. HDA group, as determined by a one-way ANOVA followed by Tukey’s post-hoc test. LDA: low-dose aripiprazole; HDA: high-dose aripiprazole; DHDA: double high-dose aripiprazole-treated groups.

ImageJ analysis of the Von Kossa-stained growth plate cartilage of the femur sections revealed a significant reduction in the length of hypertrophied chondrocytes in the HDA and DHDA-treated groups when compared to the control group (*p* < 0.05). However, such statistically significant findings were not observed among the control and LDA groups (Figure 5).

### 3.7. Effect of Aripiprazole on the Thickness of Fetal Cortical and Trabecular Bones

**Figure 6:**
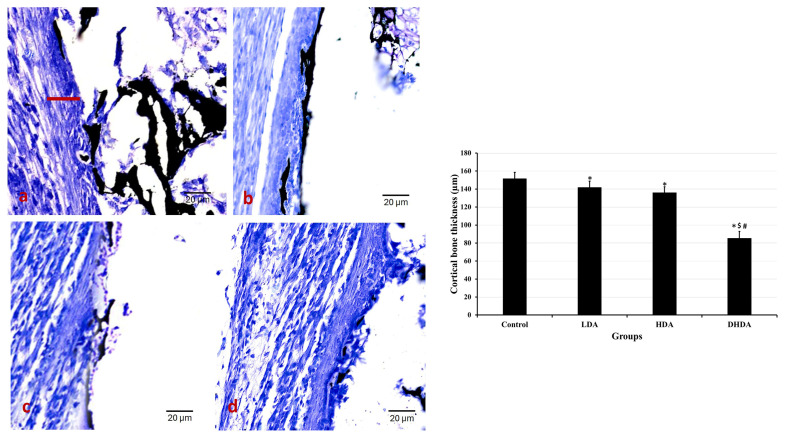
Von Kossa-stained longitudinal sections of fetal femur showing (**a**–**d**) the cortical bone thickness (µm) quantified in the (**a**) control, (**b**) LDA, (**c**) HDA, and (**d**) DHDA-treated groups. Note the significant reduction in thickness of the cortical bone among treated groups (red bar (**a**) represents the cortical bone measured in different groups). Scale bar: 20 µm. Histogram values are expressed as Mean ± SD. * *p* < 0.05 vs. control group, $ *p* < 0.05 vs. LDA group, and # *p* < 0.05 vs. HDA group, as determined by a one-way ANOVA followed by Tukey’s post-hoc test. LDA: low-dose aripiprazole; HDA: high-dose aripiprazole; DHDA: double high-dose aripiprazole-treated groups.

**Figure 7:**
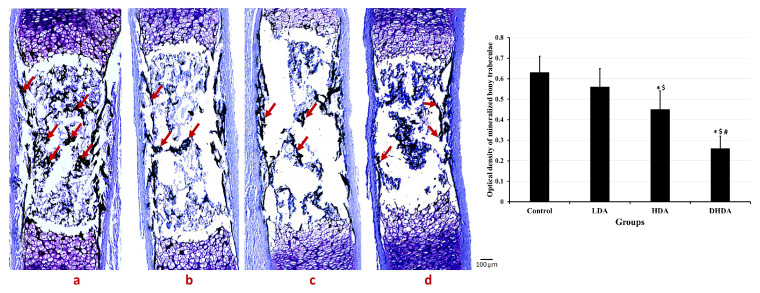
Von Kossa-stained longitudinal sections of the fetal femur showing the optical density of bony trabeculae in (**a**) control, (**b**) LDA, (**c**) HDA, and (**d**) DHDA-treated groups. Arrows are pointing to the trabecular mass quantified in the diaphysis. Note the significant reduction in trabecular mass of the diaphysis among treated groups (red arrows pointing to the trabecular bone in different groups). Length of the hypertrophied chondrocytes (µm) shown in Figure 5b–d is photographed from the histological section presented in (**b**–**d**). Cortical thickness shown in Figure 6d is photographed from the histological section presented in (**d**). Scale bar: 100 µm. Histogram values are expressed as Mean ± SD. * *p* < 0.05 vs. control group, $ *p* < 0.05 vs. LDA group, and # *p* < 0.05 vs. HDA group, as determined by a one-way ANOVA followed by Tukey’s post-hoc test. LDA: low-dose aripiprazole; HDA: high-dose aripiprazole; DHDA: double high-dose aripiprazole-treated groups.

Quantification of the thickness of fetal cortical femur from ImageJ analysis of the Von Kossa staining revealed a significant decrease in the thickness of cortical bone of the LDA, HDA, and DHDA-treated groups when compared to the control group (*p* < 0.05) (Figure 6). In addition to the cortical bone thickness, the optical density of trabecular bone measured from ImageJ analysis of the Von Kossa-stained femur sections revealed a significant decrease in the density of trabecular bone of the HDA and DHDA-treated groups when compared to the control group (*p* < 0.05) (Figure 7).

## 4. Discussion

The present experimental work was conducted to directly explore the fetal skeletal health in pregnant rats treated with aripiprazole, and findings of this study clearly reflect the teratological effects of aripiprazole on the ossification patterns by showing the complete, delayed, or absent ossification centers of the fetal appendicular skeleton in a dose-dependent manner.

The current study revealed a significant reduction in the number of completely ossified bony centers of the metacarpals, metatarsals, and hip (ilium and ischium) bones with a significant increase in the number of delayed and absent ossification centers in the LDA, HDA, and DHDA-treated groups when compared to the control group in a dose-dependent manner. In addition to these findings, the pubis presented with 0% completely ossified bony centers in almost all the groups. This typical phenomenon of our observation aligns with a normal ossification pattern of the hip bone in 20-day-old rat pups, demonstrating a sequential ossification of “*ilium-first, ischium-next and the pubis-last*” [23].

Our findings of Alizarin Red S-stained fetal skeletons revealed a statistically significant reduction in the lengths of ossified portions of the humerus, radius, ulna, femur, tibia, and fibula in the LDA, HDA, and DHDA-treated groups when compared to the control group. The fetal skeleton, particularly the appendicular skeleton, grows rapidly and increases its volumetric density during the mid-gestation period. During this period, any adverse exposure to antipsychotic drugs such as aripiprazole traverses the placental barrier and diffuses into fetal tissue by inducing significant degenerative alterations in placental morphology [13]. This phenomenon not only reduces the skeletal mass but also the FW and growth rate of the fetus [24] by increasing the oxidative stress resulting from the maternal ingestion of aripiprazole [25]. It is reported that oxidative stress stands as a cornerstone mechanism of teratogenicity, frequently disrupting normal fetal growth patterns [25]. Aripiprazole is known to strongly interfere with the function of 7-dehydrocholesterol reductase enzyme (DHCR7), which in turn leads to marked elevation of 7-dehydrocholesterol levels (7-DHC) and reduction in desmosterol (DES) across all somatic tissues [26]. DHCR7 activity is considered a vital indicator to assess prenatal toxicity, and any mutations in DHCR7 could lead to skeletal deformities as seen in Smith–Lemli–Opitz syndrome (SLOS) [27] and Fetal Fentanyl Syndrome [28].

Although aripiprazole administration reduced the ossified length of long bones in a dose-dependent manner, significant positive correlations between FW and the lengths of all ossified long bones were not noted. A statistically significant positive correlation was noted only between FW, humerus, radius, and ulna from the control group when compared to the treated groups. This indicates that under normal conditions, FW and ossified bone length increase in tandem, reflecting a synchronized and healthy developmental trajectory with a hypothesis supporting the correlation between FW and the lengths of the ossified long bones being the critical indicator of proportional skeletal development [29]. However, maternal ingestion of aripiprazole appears to fundamentally disrupt this biological harmony. In the LDA-treated group, the significant positive correlation between FW and ossified bone lengths vanished with shifting towards negative values. This trend was more pronounced in the HDA-treated group, where every ossified bone length showed a negative correlation with FW. While these negative values do not reach statistical significance, the shift from strong positives in the control group to consistent negatives in HDA and DHDA-treated groups suggests loss of predictable growth patterns, where FW no longer provides any reliable indication about the length of ossified long bones. Overall, these findings highlight that aripiprazole exposure alters the normal positive relationship between FW and skeletal growth, with increasing doses leading to a decoupling of somatic and skeletal development in rat fetuses [30].

In the current study, Alizarin Red S-stained fetal skeletons could not explain the microarchitectural changes occurring in the thickness of cortical and trabecular bones. Therefore, the femur histological sections were obtained to understand the extent of mineralization. Von Kossa-stained longitudinal sections of the femur in this study showed all the regions, right from the epiphysis, growth plate cartilage, hypertrophied chondrocytes, primary spongiosa, and diaphysis, among all groups. Hypertrophied chondrocytes are morphologically enlarged and hydrated cells that are found in the growth plate cartilage of long bones between the non-mineralized cartilage and the ossified bone [31]. The axial and appendicular bones are formed through the process of endochondral ossification. In short, endochondral ossification is a complex process that involves a series of steps, including the generation of hypertrophied chondrocytes in the growth plate cartilage and the eventual differentiation of these cells into osteoblasts forming the bony collars [32,33]. Studies have proved that these sequential steps are regulated by key transcription factors, such as Sox9, which is the main regulator of early chondrogenesis, promoting chondrocyte proliferation and differentiation, and Runx2, which is the primary regulator of chondrocyte hypertrophy [34,35]. Based on these factors, the rate of longitudinal bone growth in the growth plate is reflected by the rate of production of new cells per column and the average size of hypertrophic cell columns [36,37]. Our findings reveal that the control group pups displayed well-developed growth plates with hypertrophied chondrocytes separated by calcified bone, and the LDA, HDA, and DHDA-treated group pups presented a smaller number and reduced size of the hypertrophic chondritic layer of the growth plate cartilage in a dose-dependent manner. This reduction in the length of growth plate cartilage could be attributed to its anticholinergic effects, which may inhibit the release of growth hormone [38].

As observed from the longitudinal sections of the femur, aripiprazole inhibited the conversion of hypertrophic chondrocytes into the mineralized bony collars, and we assume this might be the mechanism through which aripiprazole delays endochondral ossification in the fetal skeleton. Since endochondral ossification is a complex process that begins in the fetus and continues through growth, with bone lengthening occurring via growth plate proliferation followed by ossification, any alteration at this stage has significant consequences on the length of bone. However, the precise molecular mechanisms behind all these events need to be thoroughly evaluated.

Histomorphometric analysis showed disorganization of bone parameters in the pups exposed to aripiprazole during the embryonic period. The thickness of the cortical and trabecular bones significantly reduced in the LDA, HDA, and DHDA-treated groups when compared to the control group. This observation may be attributed to an imbalance between osteoclastic and osteoblastic activities, resulting in incomplete bone remodeling, with reduced cortical and trabecular bone thickness from the maternal administration of aripiprazole. A recent review reveals that the AAPDs, such as aripiprazole, reduce bone density, and prolonged use seems to be associated with an increased risk of bone fragility and fractures [39]. The underlying mechanism explaining the association between AP use and reduced bone density is not clear. However, studies argue that the APs block dopamine-D2 receptors, which consequently attenuates the inhibitory action of dopamine on the prolactin secreted from the pituitary gland. This results in hyperprolactinemia, which may increase bone loss [40]. However, such mechanisms are to be explored with a thorough understanding and application of molecular studies in the future.

## 5. Conclusions

In conclusion, maternal ingestion of different doses of aripiprazole during gestation exhibited delayed ossification patterns of the appendicular skeleton. It is also evident from our histomorphometrical studies that higher doses of aripiprazole beyond the therapeutic dosage are associated with a series of serious fetal bone growth restriction patterns.

## Figures and Tables

**Table 1:** Total number of examined ossified bony centers in the appendicular skeleton of the control and aripiprazole-treated groups in 20-day rat fetuses.

Groups (No. of Fetuses)	No. of Dams	Total No. of Forelimb Bony Centers (10 × 2) ^a^	Total No. of Hindlimb Bony Centers (11 × 2) ^b^
Control (39)	10	780	858
LDA (38)	10	760	836
HDA (45)	10	900	990
DHDA (29)	10	580	638

^a^ Number of forelimb bony centers (10 in each forelimb: scapula, clavicle, humerus, radius, ulna, and 5 metacarpals). ^b^ Number of hindlimb bony centers (11 in each hindlimb: ilium, ischium, pubis, femur, tibia, fibula, and 5 metatarsals).

**Table 2:** Effect of maternal ingestion of aripiprazole on ossification centers of metacarpals and metatarsals in 20-day-old rat fetuses.

Groups (No. of Fetuses)	No. of Dams	Metacarpals (5 × 2)		Metatarsals (5 × 2)	
		Complete	Delayed/Absent	Complete	Delayed/Absent
Control (39)	10	144 (36.92%) 14.4 ± 6.23	246 (63.08%) 24.6 ± 11.51	148 (37.94%) 14.8 ± 7.28	242 (62.05%) 24.2 ± 11.44
LDA (38)	10	64 (16.84%) 6.4 ± 4.08 ^a^	316 (83.15%) 31.6 ± 7.98 ^a^	50 (13.15%) 5.0 ± 3.91 ^a^	330 (86.84%) 33.0 ± 6.81 ^a^
HDA (45)	10	94 (20%) 9.4 ± 4.01 ^a^	356 (79.11%) 35.6 ± 8.52 ^a^	74 (16.44%) 7.4 ± 5.50 ^a^	376 (83.55%) 37.6 ± 10.86 ^a^
DHDA (29)	10	36 (12.41%) 3.6 ± 2.15 ^a,b^	254 (87.58%) 25.4 ± 7.90 ^a,b^	34 (11.72%) 3.4 ± 2.50 ^a^	256 (88.27%) 25.6 ± 7.59 ^a^

^a^ *p* < 0.01 compared to the control group. ^b^ *p* < 0.01 compared to the HDA group. Mean ± SD values were calculated using the dam as a baseline statistical unit with one-way ANOVA followed by Dunnett’s post-hoc test.

**Table 3:** Mean length (mm) of the ossified portions of the long bones of the forelimbs and hindlimbs and FW (gm) of 20-day rat fetuses in the control and aripiprazole-treated groups.

Growth Parameters	Control (Mean ± SD)	LDA (Mean ± SD)	HDA (Mean ± SD)	DHDA (Mean ± SD)
Humerus	2.18 ± 0.46	2.00 ± 0.17 ^a^	1.84 ± 0.15 ^a,b^	1.58 ± 0.15 ^a,b,c^
Radius	2.06 ± 0.20	1.93 ± 0.20 ^a^	1.77 ± 0.19 ^a,b^	1.57 ± 0.18 ^a,b,c^
Ulna	1.63 ± 0.24	1.52 ± 0.23	1.46 ± 0.27 ^a^	1.32 ± 0.21 ^a,b^
Femur	1.56 ± 0.19	1.37 ± 0.19 ^a^	1.32 ± 0.28 ^a^	1.12 ± 0.30 ^a,b,c^
Tibia	1.77 ± 0.23	1.51 ± 0.21 ^a^	1.46 ± 0.29 ^a^	1.21 ± 0.36 ^a,b,c^
Fibula	1.49 ± 0.24	1.25 ± 0.24 ^a^	1.19 ± 0.31 ^a^	0.933 ± 0.25 ^a,b,c^
FW	2.23 ± 0.24	1.90 ± 0.14	1.75 ± 2.10 *	1.60 ± 0.14 *

ANOVA test followed by the Bonferroni test. Values are expressed as Mean ± SD. ^a^ *p* < 0.001 vs. control group, ^b^ *p* < 0.001 vs. LDA group, ^c^ *p* < 0.001 vs. HDA group, and * *p* < 0.05 vs. control group. LDA: low-dose aripiprazole; HDA: high-dose aripiprazole; DHDA: double high-dose aripiprazole-treated groups. FW: fetal weight.

**Table 4:** Effect of maternal ingestion of aripiprazole on ossification centers of hip bones (ilium, ischium, and pubis) in 20-day-old rat fetuses.

Groups (No. of Fetuses)	No. of Dams	Ilium (*n* = 2)		Ischium (*n* = 2)		Pubis (*n* = 2)		Total (*n* = 6)	
		Complete	Delayed/Absent	Complete	Delayed/Absent	Complete	Delayed/Absent	Complete	Delayed/Absent
Control (39)	10	76 (97.43%) 7.6 ± 2.07	2 (2.56%) 0.2 ± 0.63	48 (61.53%) 4.8 ± 3.15	30 (38.46%) 3.0 ± 3.16	0(0%)0	78 (100%) 7.8 ± 2.20	124 (52.99%) 12.4 ± 4.59	110 (47.1%) 11.0 ± 4.92
LDA (38)	10	58 (76.31%) 5.8 ± 3.19 ^a^	18 (23.68%) 1.8 ± 2.57 ^a^	16 (21.1%) 1.6 ± 2.07 ^a^	60 (78.9%) 6.0 ± 2.83 ^a^	0(0%)0	76 (100%) 7.6 ± 1.58	74 (32.45%) 7.4 ± 4.22 ^a^	154 (67.54%) 15.4 ± 5.08 ^a^
HDA (45)	10	74 (82.22%) 7.4 ± 1.90 ^a^	16 (17.77%) 1.6 ± 2.07 ^a^	30 (33.3%) 3.0 ± 2.71 ^a^	60 (66.6%) 6.0 ± 3.13 ^a^	4 (4.45%) 0.4 ± 0.84	86 (95.6%) 8.6 ± 1.35	108 (40%) 10.8 ± 4.2 ^a^	162 (60%) 16.2 ± 5.5 ^a^
DHDA (29)	10	28 (48.27%) 2.8 ± 2.35 ^a,b,c^	30 (51.72%) 3.0 ± 2.54 ^a,b,c^	6 (10.34%) 0.6 ± 0.97 ^a,c^	52 (89.65%) 5.2 ± 1.40 ^a,c^	0(0%)0	58 (100%) 5.8 ± 1.48	34 (19.5%) 3.4 ± 3.13 ^a,b,c^	140 (80.5%) 14 ± 4.71 ^a,b,c^

^a^ *p* < 0.01 compared to the control group. ^b^ *p* < 0.01 compared to the LDA group. ^c^ *p* < 0.01 compared to the HDA group. Mean ± SD values were calculated using the dam as a baseline statistical unit with one-way ANOVA followed by Dunnett’s post-hoc test.

**Table 5:** Correlations between FW and lengths of the ossified portions of the long bones of the forelimb and hindlimbs in 20-day-old rat fetuses following maternal ingestion of aripiprazole.

Groups	GP	Hu	R	U	Fe	T	Fi	FW
Control	Hu	1						
	R	0.855 **	1					
	U	0.458 *	0.667 **	1				
	Fe	0.381	0.629 **	0.587 **	1			
	T	0.559 **	0.727 **	0.597 **	0.736 **	1		
	Fi	0.325	0.637 **	0.655 **	0.823 **	0.859 **	1	
	FW	0.629 **	0.537 **	0.416 *	0.153	0.253	0.103	1
LDA	Hu	1						
	R	0.828 **	1					
	U	0.820 **	0.899 **	1				
	Fe	0.675 **	0.715 **	0.614 **	1			
	T	0.846 **	0.866 **	0.823 **	0.596 **	1		
	Fi	0.744 **	0.768 **	0.728 **	0.713 **	0.822 **	1	
	FW	−0.022	−0.080	−0.172	0.022	−0.227	−0.259	1
HDA	Hu	1						
	R	0.732 **	1					
	U	0.574 **	0.879 **	1				
	Fe	0.510 *	0.752 **	0.772 **	1			
	T	0.520 *	0.775 **	0.843 **	0.816 **	1		
	Fi	0.677 **	0.812 **	0.808 **	0.845 **	0.837 **	1	
	FW	−0.345	−0.271	−0.222	−0.334	−0.154	−0.295	1
DHDA	Hu	1						
	R	0.909 **	1					
	U	0.749 **	0.819 **	1				
	Fe	0.373	0.467	0.544 *	1			
	T	0.337	0.461	0.561 *	0.945 **	1		
	Fi	0.190	0.334	0.433	0.922 **	0.962 **	1	
	FW	0.126	0.040	0.030	0.104	−0.014	−0.046	1

GP: growth parameters; lengths of Hu: humerus; R: radius; U: ulna; Fe: femur; T: tibia; Fi: fibula; FW: fetal weight. * *p* < 0.05; ** *p* < 0.01 (Pearson correlation coefficient r).

## Data Availability

The data presented in this study are available on request from the corresponding author due to privacy and ethical reasons.

## Abbreviations

The following abbreviations are used in this manuscript:

APDs	antipsychotic drugs
BMD	bone mineral density
AAPD	atypical antipsychotic drug
AAPDs	atypical antipsychotic drugs
LDA	low-dose aripiprazole
HDA	high-dose aripiprazole
DHDA	double high-dose aripiprazole
MRTD	maximum recommended human therapeutic dose
ANOVA	analysis of variance
SPSS	Statistical Package for the Social Sciences
SD	standard deviation
DHCR7 7	dehydrocholesterol reductase enzyme
7-DHC 7	dehydrocholesterol levels
DES	desmosterol
SLOS	Smith–Lemli–Opitz syndrome
